# The relationship between intraoperative cerebral oximetry and postoperative delirium in patients undergoing off-pump coronary artery bypass graft surgery: a retrospective study

**DOI:** 10.1186/s12871-020-01180-x

**Published:** 2020-11-14

**Authors:** Leerang Lim, Karam Nam, Seohee Lee, Youn Joung Cho, Chan-Woo Yeom, Sanghyup Jung, Jung Yoon Moon, Yunseok Jeon

**Affiliations:** 1 Department of Anesthesiology and Pain Medicine, Seoul National University Hospital, 101, Daehak-ro, Jongno-gu, Seoul, Republic of Korea 03080; 2 Department of Neuropsychiatry, Seoul National University Hospital, 101, Daehak-ro, Jongno-gu, Seoul, Republic of Korea 03080

**Keywords:** Off-pump coronary artery bypass graft surgery, Cerebral oximetry, Delirium

## Abstract

**Background:**

Cerebral oximetry has been widely used to measure regional oxygen saturation in brain tissue, especially during cardiac surgery. Despite its popularity, there have been inconsistent results on the use of cerebral oximetry during cardiac surgery, and few studies have evaluated cerebral oximetry during off pump coronary artery bypass graft surgery (OPCAB).

**Methods:**

To evaluate the relationship between intraoperative cerebral oximetry and postoperative delirium in patients who underwent OPCAB, we included 1439 patients who underwent OPCAB between October 2004 and December 2016 and among them, 815 patients with sufficient data on regional cerebral oxygen saturation (rSO_2_) were enrolled in this study. We retrospectively analyzed perioperative variables and the reduction in rSO_2_ below cut-off values of 75, 70, 65, 60, 55, 50, 45, 40, and 35%. Furthermore, we evaluated the relationship between the reduction in rSO_2_ and postoperative delirium.

**Results:**

Delirium occurred in 105 of 815 patients. In both univariable and multivariable analyses, the duration of rSO_2_ reduction was significantly longer in patients with delirium at cut-offs of < 50 and 45% (for every 5 min, adjusted odds ratio (OR) 1.007 [95% Confidence interval (CI) 1.001 to 1.014] and adjusted OR 1.012 [1.003 to 1.021]; *p* = 0.024 and 0.011, respectively). The proportion of patients with a rSO_2_ reduction < 45% was significantly higher among those with delirium (adjusted OR 1.737[1.064 to 2.836], *p* = 0.027).

**Conclusions:**

In patients undergoing OPCAB, intraoperative rSO_2_ reduction was associated with postoperative delirium. Duration of rSO_2_ less than 50% was 40% longer in the patients with postoperative delirium. The cut-off value of intraoperative rSO_2_ that associated with postoperative delirium was 50% for the total patient population and 55% for the patients younger than 68 years.

**Supplementary information:**

**Supplementary information** accompanies this paper at 10.1186/s12871-020-01180-x.

## Background

Cerebral oximetry has been widely used to measure regional oxygen saturation in brain tissue continuously and non-invasively, especially during general anesthesia [1]. Using near-infrared spectroscopy (NIRS), cerebral oximetry measures regional cerebral oxygen saturation (rSO_2_) by analyzing the different intensities of light at specific wavelengths transmitted and received [2, 3] and monitor rSO_2_ underlying frontal lobes, which are vulnerable to hypoxic and hypotensive injury [4].

Because the neurological outcome is still a matter of concern in cardiac surgery, cerebral oximetry-based resuscitation during cardiac surgery has been increasingly adopted by anesthesiologists [5]. Among post-cardiac surgery neurologic complications, the reported prevalence of delirium is from 3.1% up to 52% by population and diagnostic methods, with higher prevalence in older population and aortic surgery patients, and more detection with precise cognitive function test by highly trained personnel [6–9]. Moreover, delirium is known to prolong intensive care unit and hospital stays, increase morbidity and mortality, and reduce cognitive and functional recovery [10–12]. Thus, among neurologic complications, delirium is a serious and relatively common neurologic complication.

Despite the widespread use of cerebral oximetry, there have been inconsistent results regarding the relationship between the intraoperative use of cerebral oximetry and improved postoperative neurologic outcomes in cardiac surgery patients [13–17]. There have been few trials designed to identify the optimal cut-off values for cerebral oximetry, resulting in various criteria being used by different studies. Moreover, few studies on cerebral oximetry in patients undergoing off-pump coronary artery bypass graft surgery (OPCAB) have been carried out.

To evaluate the relationship between the intraoperative cerebral oximetry and postoperative delirium and identify the optimal cut-off values for intraoperative cerebral oximetry during OPCAB, we retrospectively analyzed data of intraoperative cerebral oximetry values and postoperative delirium from patients who underwent OPCAB.

## Methods

### Study population and anesthetic methods

This was a retrospective single-center study approved by the Institutional Review Board of Seoul National University Hospital (IRB no. 1702–114-833). The requirement for written informed consent was waived. After IRB approval, we reviewed the electronic medical records of all patients aged over 18 years who had undergone coronary artery bypass graft surgery (CABG) between October 2004 and December 2016. Among them, we included only patients who had isolated OPCAB under general anesthesia. Patients who had been supported with perioperative intra-aortic balloon pump and/or extracorporeal membrane oxygenation were also excluded. Patients with insufficient rSO_2_ records less than 10 times, the − 2 standard deviations (SDs) of the times of rSO_2_ measurement were excluded.

During the period, anesthesia for OPCAB was performed as per the institutional routine protocol at that period. When the patients entered the operating room, bi-hemispheric rSO_2_ was measured by NIRS from the forehead in the supine position, with other types of monitoring used for vital signs. We used INVOS Cerebral Oximeters (Medtronic, MN, USA) for rSO_2_ measurement. Every drug used during anesthesia was given intravenously. Patients were also monitored with a Swan-Ganz catheter (Edwards Lifesciences, Irvine, CA, USA) for mixed venous oxygen saturation (SvO_2_) and cardiac index (C.I.). Patients were transferred to the cardio-pulmonary intensive care unit (ICU) after surgery being sedated and intubated.

### Data collection and definition

Baseline characteristics and perioperative variables known to be related to delirium after cardiac surgery were collected [6, 9, 18–23]. These included age, sex, American Society of Anesthesiologists (ASA) classification, order of surgery, emergency, operation year, underlying diseases such as hypertension, diabetes mellitus, dyslipidemia, atrial fibrillation, history of myocardial infarction or stroke, and laboratory variables like left ventricular ejection fraction (EF), hematocrit, serum creatinine, estimated glomerular filtration rate (eGFR), serum albumin, and C-reactive protein. Postoperative outcomes, including ICU and hospital lengths of stay, acute kidney injury, new-onset atrial fibrillation, reintubation rate, and in-hospital death, were also collected.

Intraoperative variables included total anesthesia and operation times. We also used the electronic anesthetic record to extract the mean arterial pressure (MAP), SvO_2_, C.I., and bi-hemispheric rSO_2_, independently, every 5 min. The resting MAP before anesthesia induction and initially measured SvO_2_ and C.I. were used as baseline values. The MAPs were recorded automatically by the anesthetic monitor, while other variables were recorded manually every 5 to 15 min. We conducted data pre-processing on these variables according to the following steps using R (R3.5.1; The R Foundation for Statistical Computing). First, we excluded patients who had rSO_2_ records that included fewer than ten measurements. Second, all data exceeding − 2 SDs and + 2 SDs for each variable were considered abnormally recorded and removed. Third, empty values for data recorded at 5-min intervals were substituted by the mean of the nearest two records.

After these substitutions, we calculated the total time for which the rSO_2_ values decreased below each cut-off (75, 70, 65, 60, 55, 50, 45, 40, and 35% of the absolute values). We also treated the reduction in rSO_2_ for at least one measurement below each cut-off written above as a categorical variable. The same substitutions and time calculations were carried out for C.I., SvO_2_, and MAP, and mean values were used for receiver operating characteristic (ROC) analysis.

Postoperative delirium was determined by institutional neuropsychiatrists (C-W Yeom and colleagues) on the basis of electronic medical records. Neuropsychiatrists reviewed the doctors’ records and nursing records, including the Confusion Assessment Method (CAM) for ICU (CAM-ICU) [24–26] score evaluated by the attending nurse in the ICU, consultations with neuropsychiatrists and neurologists, and prescriptions for drugs that could be used for delirium (e.g., haloperidol or quetiapine). According to Diagnostic and Statistical Manual of Mental Disorders-5 (DSM-5) [27] and Short-CAM [28] criteria, the neuropsychiatrists evaluated the signs and symptoms recorded and determined whether or not the patient had undergone postoperative delirium.

### Statistical analysis

All statistical analyses were performed using SPSS, version 23.0, for Windows (IBM Corp., Armonk, NY, USA). We hypothesized a normal distribution for all variables. All categorical variables were analyzed using chi-square tests or Fisher’s exact test. All continuous variables were analyzed using Student’s *t*-test and logistic regression analysis. A *p-*value *<* 0.05 was considered statistically significant.

First, we conducted a univariable analysis for all variables collected. A *p*-value *<* 0.10 was used to select significant predictors for multivariable analysis. Next, a multivariable logistic regression analysis was performed with selected variables, and total times of rSO_2_ under each cut-off using a backward stepwise method. We compared the predictive ability of each prediction model to identify significant cut-offs for rSO_2_ related to delirium after off-pump coronary artery bypass.

## Results

**Fig. 1:**
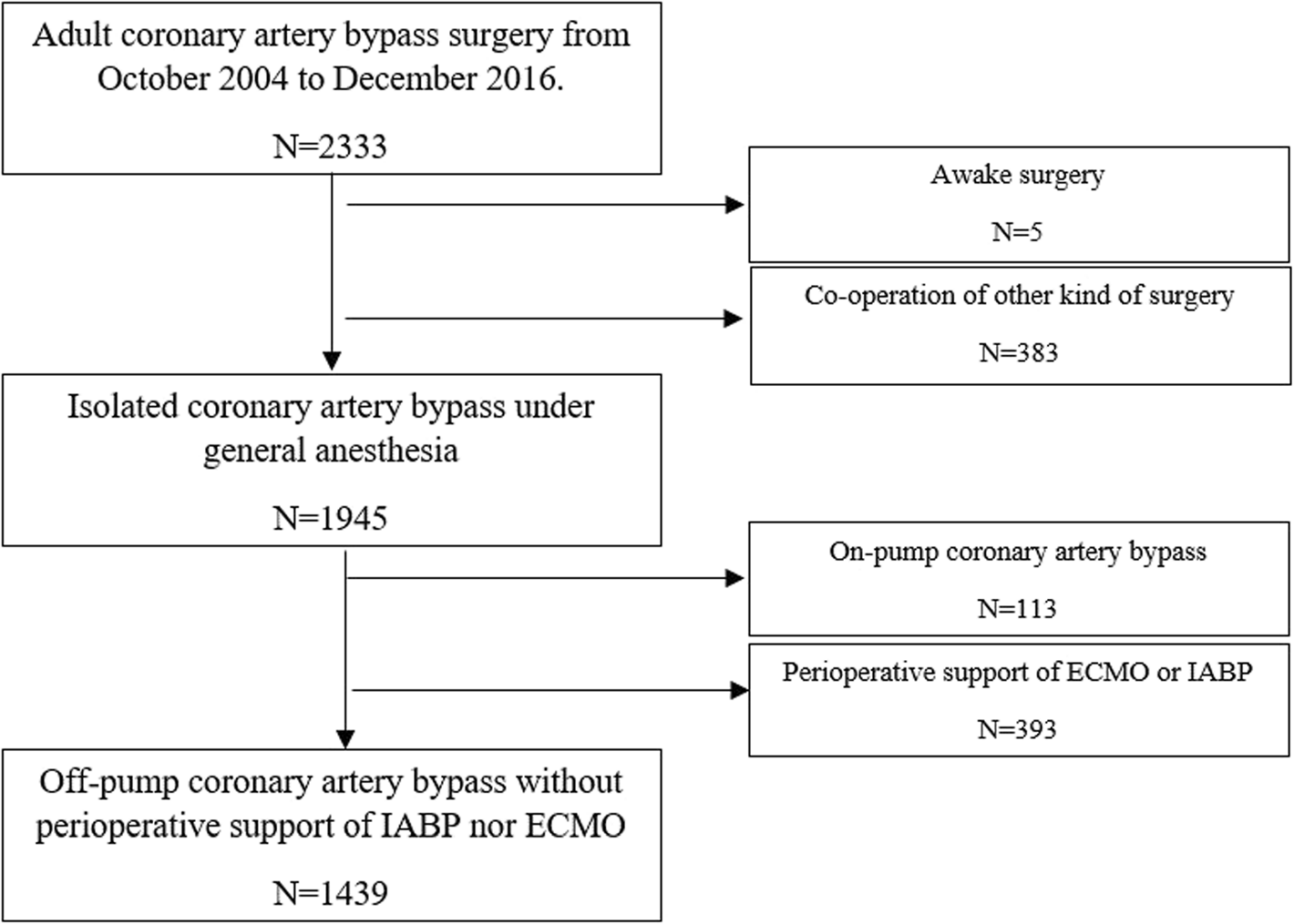
Flow chart for patient selection

During the study period, 2333 patients underwent CABG. 1945 patients were undergone isolated CABG with general anesthesia. We also excluded 506 patients because of the perioperative support of IABP or ECMO, or intraoperative use of CPB. Finally, 1439 patients were included in the study. After data pre-processing, 815 patients in total were included. The flow chart for patient selection is shown in Fig. 1.

The baseline and perioperative characteristics of the patients are shown in Table 1 (no delirium group vs. delirium group, 710 [87.1%] vs. 105 [12.9%] patients). The delirium group had a higher average age and C-reactive protein level, and lower hematocrit, eGFR, and albumin readings, and more underlying hypertension. The group also had longer ICU and hospital stays and more frequent postoperative acute kidney injury and new onset atrial fibrillation and reintubation. In-hospital deaths numbered 3 (2.9%) in the delirium group and 0 in the no delirium group, but this did not reach the level of statistical significance.

**Table 1:** Baseline and perioperative characteristics of patients with or without delirium

Characteristics	No delirium(*n* = 710)	Delirium(*n* = 105)	*P*-values
Patients characteristics			
Age (year)	65.2 ± 9.6	71.9 ± 8.2	< 0.001
Male sex	556 (78.3%)	74 (70.5%)	0.08
BMI (kg/m^2)^	24.6 ± 3.3	24.1 ± 3.1	0.13
ASA physical status			0.32
1	19 (2.7%)	1 (1.0%)	
2	200 (28.2%)	25 (23.8%)	
3	478 (67.3%)	75 (71.4%)	
4	13 (1.8%)	4 (3.8%)	
Hypertension	456 (64.2%)	83 (79.0%)	0.003
Diabetes mellitus	349 (49.2%)	57 (54.3%)	0.33
Dyslipidemia	268 (37.7%)	35 (33.3%)	0.38
Myocardial infarction	80 (11.3%)	15 (14.3%)	0.37
Atrial fibrillation	50 (7.0%)	7 (6.7%)	0.89
Chronic kidney disease	275 (38.7%)	43 (41.0%)	0.66
History of stroke	452 (63.7%)	64 (63.3%)	0.59
Left ventricle ejection fraction (%)	55.1 ± 11.1	53.9 ± 12.5	0.30
Hematocrit (%)	34.8 ± 4.0	33.9 ± 4.1	0.03
Creatinine (mg/dL)	1.4 ± 1.7	1.5 ± 1.7	0.3
Estimated GFR (ml/min/1.73m^2^)	73.9 ± 27.3	63.5 ± 26.7	< 0.001
Albumin (g/dL)	4.0 ± 0.4	3.8 ± 0.4	< 0.001
C-reactive protein (mg/dL)	0.7 ± 1.4	1.1 ± 2.3	0.006
Intraoperative variables			
Operation duration (min)	362.2 ± 53.4	362.6 ± 61.7	0.95
Anesthesia duration (min)	438.1 ± 54.6	436.1 ± 68.2	0.77
Re-do operation	7 (1.0%)	1 (1.0%)	0.97
Emergency	76 (10.7%)	12 (11.4%)	0.82
Op year			0.602
2005–2009	54	6	
2010–2014	433	69	
2015-	223	30	
Postoperative medical status			
ICU lengths of stay (days)	2.3 ± 1.7	5.8 ± 7.1	< 0.001
Hospital lengths of stay (days)	9.9 ± 7.1	22.1 ± 25.3	< 0.001
Acute kidney injury	133 (18.7%)	34 (32.4%)	0.001
New onset atrial fibrillation	146 (20.6%)	31 (29.5%)	0.04
Reintubation	27 (3.8%)	18 (17.1%)	< 0.001
In-hospital death	0	3 (2.9%)	

The values are expressed as mean ± standard deviation or number (%). *ASA* American Society of Anesthesiologists, *BMI* Body mass index, *GFR* Glomerular filtration rate

The duration and number of intraoperative rSO_2_ measurements below each cut-off are shown in Table 2. The duration of rSO_2_ reduction was significantly longer in patients with delirium for the cut-offs of < 50 and 45% (for each group, mean duration (SD) of 138.7(202.7) and 64.6(141.5) vs. 100.9(159.6) and 39.3(100.6), *p =* 0.031 and 0.027, respectively). There was a significantly higher proportion of patients with an rSO_2_ reduction < 45% among those with delirium (for each group, number of patients (%) of 228(32.1) vs. 44(41.9), *p =* 0.048)*.* We also calculated number needed to treat (NNT) for the cut-off of 45%. According to the Table 2, the control event rate (CER) for delirium is 44/(44 + 228) = 0.16 and the experimental event rate (EER) for delirium is 61/(61 + 482) = 0.11. The absolute risk reduction (ARR) = CER-EER = 0.16–0.11 = 0.05, therefore the NNT is 1/(ARR) = 1/0.05 = 20. That is, 20 patients need to be treated to reduce 1 episode of delirium.

**Table 2:** Comparison of intraoperative rSO_2_ between delirium and no delirium group

rSO_2_	No delirium(*n* = 710)	Delirium(*n* = 105)	*P*-values
Mean; %	55.5 ± 6.8	54.8 ± 7.74	0.32
Minimum; %	47.6 ± 8.1	46.7 ± 8.33	0.30
Mean duration of rSO_2_ reduction; min			
< 75%	451.0 ± 141.7	468.0 ± 175.7	0.27
< 70%	442.0 ± 147.9	459.9 ± 182.1	0.28
< 65%	402.3 ± 167.4	418.9 ± 195.4	0.36
< 60%	318.2 ± 193.4	341.9 ± 230.9	0.25
< 55%	204.1 ± 196.1	231.0 ± 230.3	0.20
< 50%	100.9 ± 159.6	138.7 ± 202.7	0.03
< 45%	39.3 ± 100.6	64.6 ± 141.5	0.03
< 40%	11.7 ± 49.2	18.3 ± 82.1	0.26
< 35%	4.0 ± 28.9	7.1 ± 50.7	0.38
Number of patients with rSO_2_ reduction			
< 70%	709 (99.9%)	105 (100%)	1
< 65%	703 (99.0%)	104 (99.0%)	0.97
< 60%	669 (94.2%)	98 (93.3%)	0.72
< 55%	573 (80.7%)	84 (80.0%)	0.87
< 50%	407 (57.3%)	69 (65.7%)	0.11
< 45%	228 (32.1%)	44 (41.9%)	0.048
< 40%	108 (15.2%)	17 (16.2%)	0.80
< 35%	41 (5.8%)	6 (5.7%)	0.98

The values are expressed as mean ± standard deviation for mean, minimum rSO_2_ and mean duration of rSO_2_ reduction, number (%) for the incidence of rSO_2_ reduction. *rSO*_*2*_ Regional cerebral oxygen saturation

Intraoperative hemodynamic variables are shown in Supplementary Table 1 in Additional file 1. Based on the results of an ROC analysis for the mean values of each variable, the cut-off was determined as 68 mmHg, 2.2 L/min/m^2^, and 64% for MAP, C.I., and SvO_2_, respectively. The total durations of reduction below the cut-off and minimum values were calculated. For all three variables, the total duration of reduction below each cut-off was significantly longer in the delirium group than the no delirium group (*p =* 0.001), and these cut-off values were selected for a multivariable analysis as categorical variables.

The odds ratio (OR), 95% confidence interval (CI), and *p-*values of rSO_2_ for each cut-off are shown in Table 3. The OR and 95% CI were calculated for every 5 min of rSO_2_ reduction below each cut-off value. Age, sex, hypertension, preoperative hematocrit, eGFR, serum albumin and C-reactive protein level, intraoperative MAP, C.I. and SvO_2_ reduction below each cut-off of ROC analysis were considered as covariables. There was no multicollinearity between the variables included in the analysis, especially between the intraoperative hemodynamic variables and rSO_2_ for the occurrence of postoperative delirium. Multivariable logistic regression analysis revealed that the duration of rSO_2_ below the 50 and 45% cut-offs was significantly associated with postoperative delirium (for every 5 min, adjusted OR 1.007 [95% CI 1.001–1.014] and 1.012 [1.003–1.021]; *p =* 0.024 and 0.011, respectively). Each model showed good fitness (Hosmer-Lemeshow’s goodness-of-fit: *p =* 0.729 and 0.962, respectively). The rSO_2_ values below 45% for at least one measurement were significantly associated with postoperative delirium, and the model fitness was good (adjusted OR 1.737, *p =* 0.027; Hosmer-Lemeshow’s goodness-of-fit: *p =* 0.923; Table 4). According to the analysis of variance between the two group for the duration of rSO_2_ reduction < 45% or 50%, and delirium, the *p*-values of linearity were < 0.05 and the *p*-values of deviation from linearity were > 0.05, indicating that there is a linear relationship between the duration of rSO_2_ reduction below 45% or 50% and the probability of postoperative delirium. The duration of rSO_2_ below 50 and 45% was also associated with postoperative acute kidney injury, a longer ICU stay, and longer hospital stay (Supplementary Table 2 in Additional file 1).

**Table 3:** Unadjusted and adjusted odds ratios of intraoperative reduction of rSO_2_ of each cut-offs for postoperative delirium

Intraoperative rSO_2_	Unadjusted OR (95% CI)	*P*-values	Adjusted OR (95% CI)	*P*-values
Mean	0.985 (0.957 to 1.014)	0.32	0.976 (0.942 to 1.011)	0.18
Minimum	0.987 (0.962 to 1.012)	0.30	0.977 (0.948 to 1.006)	0.12
Duration of rSO_2_ reduction (for every 5 min)				
< 75%	1.004 (0.997 to 1.010)	0.27	1.006 (0.999 to 1.013)	0.12
< 70%	1.004 (0.997 to 1.010)	0.28	1.005 (0.998 to 1.012)	0.14
< 65%	1.003 (0.997 to 1.009)	0.36	1.004 (0.997 to 1.011)	0.24
< 60%	1.003 (0.998 to 1.008)	0.25	1.004 (0.998 to 1.010)	0.16
< 55%	1.003 (0.998 to 1.008)	0.20	1.004 (0.999 to 0.010)	0.15
< 50%	1.006 (1.001 to 1.011)	0.03	1.007 (1.001 to 1.014)	0.02
< 45%	1.009 (1.001 to 1.017)	0.03	1.012 (1.003 to 1.021)	0.01
< 40%	1.009 (0.994 to 1.025)	0.26	1.013 (0.995 to 1.030)	0.15
< 35%	1.011 (0.986 to 1.037)	0.38	1.021 (0.990 to 1.053)	0.19
Occurrence of rSO_2_ reduction				
< 70%	.	1	.	1
< 65%	1.036 (0.126 to 8.502)	0.97	.	1
< 60%	0.858 (0.374 to 1.966)	0.72	1.460 (0.423 to 5.044)	0.55
< 55%	0.956 (0.572 to 1.598)	0.87	0.935 (0.492 to 1.777)	0.84
< 50%	1.427 (0.929 to 2.192)	0.11	1.599 (0.965 to 2.649)	0.07
< 45%	1.525 (1.003 to 2.317)	0.048	1.737 (1.064 to 2.836)	0.03
< 40%	1.077 (0.616 to 1.882)	0.80	1.236 (0.657 to 2.326)	0.51
< 35%	0.989 (0.409 to 2.390)	0.98	0.839 (0.306 to 2.299)	0.73

*rSO*_*2*_ Regional cerebral oxygen saturation, *OR* Odds ratio, *CI* Confidence interval

**Table 4:** Odds ratios of predictors for postoperative delirium

Variables	Multivariable logistic regression – OR (95% CI)	Univariable logistic regression – OR (95% CI)
Age (year)	1.093 (1.058 to 1.129)	1.097 (1.066 to 1.128)
Sex (Female)	–	1.512 (0.959 to 2.386)
Preoperative		
Hypertension	1.908 (1.062 to 3.428)	2.101 (1.282 to 3.445)
Hematocrit (%)	–	0.943 (0.896 to 0.993)
estimated GFR (ml/min^/^1.73m^2^)	–	0.987 (0.980 to 0.994)
Albumin (g/dL)	0.485 (0.276 to 0.852)	0.384 (0.244 to 0.605)
C-reactive protein (mg/dL)	–	1.163 (1.044 to 1.295)
Intraoperative		
MAP < 68 mmHg	–	1.002 (1.001 to 1.004)
C.I. < 2.2 L/min^/^m^2^	–	1.002 (1.001 to 1.003)
SvO_2_ < 64%	–	1.003 (1.001 to 1.005)
Occurrence of rSO_2_ < 45%	1.737 (1.064 to 2.836)	1.525 (1.003 to 2.317)

*OR* Odds ratio, *CI* Confidence interval, *GFR* Glomerular filtration rate, *MAP* Mean arterial pressure, *C.I.* Cardiac index, *SvO*_*2*_ Mixed venous oxygen saturation, *rSO*_*2*_ Regional cerebral oxygen saturation

A post hoc power analysis was performed for the occurrence of rSO2 reduction < 45% with chi-squared test based on the result described in Table 2. The analysis revealed a power of this study above 0.95 for the occurrence of rSO2 reduction < 45%.

Based on the ROC analysis, the cut-off age for postoperative delirium occurrence was 68. We conducted a subgroup analysis based on this cut-off. Among 815 patients, 398 (48.8%) were under age 68, and delirium occurred in 19 patients (4.8%). Baseline and perioperative characteristics, including intraoperative hemodynamic variables, are shown in Supplementary Table 3 in Additional file 1. Based on a univariable analysis, preoperative EF, and albumin and C-reactive protein levels were selected for a multivariable analysis. Supplementary Table 4 Additional file 1 shows the duration and number of intraoperative rSO_2_ values below each cut-off in patients under 68 years of age. The mean and minimum rSO_2_ values were significantly lower in the delirium group. The duration of rSO_2_ reduction was significantly longer in patients with delirium for the cut-offs of < 55, 50, and 45%, and the proportion of patients with an rSO_2_ reduction below 50 and 45% was significantly higher among those with delirium. These cut-offs were higher than those of the overall group in Table 2. In the multivariable logistic regression analysis, the duration of rSO_2_ lower than 55, 50, and 45% was significantly associated with postoperative delirium (for every 5 min, adjusted OR 1.012, 1.015, and 1.015, *p =* 0.035, 0.006, and 0.024, respectively), as shown in Table 5. However, the model fitness for the cut-off of 55% was not good (Hosmer-Lemeshow’s goodness-of-fit: *p =* 0.022), whereas those for the other cut-offs were good. The area under receiver operating characteristic (AUROC) for prediction models for patients under 68 years of age are shown in Additional file 2. The AUROC for the model without rSO_2_ was 0.688 (95% CI 0.565–0.816, *p =* 0.007), and improved with rSO_2_ measurement, up to 0.752 (95% CI 0.640–0.865, *p <* 0.001) with the duration of rSO_2_ < 50%.

**Table 5:** Odds ratios of intraoperative reduction of rSO_2_ of each cut-offs for postoperative delirium in patients under age 68

Intraoperative rSO_2_	Unadjusted OR (95% CI)	*P*-values	Adjusted OR (95% CI)	*P*-values
Mean	0.920 (0.869 to 0.975)	0.004	0.927 (0.874 to 0.984)	0.01
Minimum	0.934 (0.886 to 0.984)	0.01	0.940 (0.891 to 0.992)	0.03
Duration of rSO_2_ reduction (for every 5 min)				
< 75%	0.999 (0.982 to 1.016)	0.87	0.997 (0.979 to 1.016)	0.78
< 70%	1.001 (0.985 to 1.016)	0.95	0.999 (0.982 to 1.016)	0.89
< 65%	1.005 (0.992 to 1.018)	0.48	1.003 (0.988 to 1.017)	0.73
< 60%	1.008 (0.997 to 1.019)	0.16	1.005 (0.993 to 1.018)	0.36
< 55%	1.011 (1.001 to 1.022)	0.03	1.012 (1.001 to 1.022)	0.04
< 50%	1.015 (1.005 to 1.025)	0.004	1.015 (1.004 to 1.025)	0.006
< 45%	1.016 (1.003 to 1.029)	0.02	1.015 (1.002 to 1.029)	0.02
< 40%	1.014 (0.987 to 1.042)	0.3	1.010 (0.982 to 1.039)	0.49
< 35%	1.017 (0.980 to 1.057)	0.37	1.011 (0.972 to 1.052)	0.59
Occurrence of rSO_2_ reduction				
< 75%	.	1	.	1
< 70%	.	1	.	1
< 65%	.	1	.	1
< 60%	.	1	.	1
< 55%	4.970 (0.654 to 37.782)	0.12	4.231 (0.551 to 32.480)	0.17
< 50%	4.156 (1.191 to 14.503)	0.03	4.013 (1.112 to 14.482)	0.03
< 45%	2.634 (1.034 to 6.709)	0.04	2.283 (0.906 to 6.266)	0.08
< 40%	2.662(0.971 to 7.295)	0.06	2.757(0.980 to 7.757)	0.06
< 35%	1.114(0.141 to 8.817)	0.92	0.989(0.118 to 8.300)	0.99

*OR* Odds ratio, *CI* Confidence interval, *rSO*_*2*_ Regional cerebral oxygen saturation

Among 417 patients over 68 years of age, the incidence of delirium was 20.6% (86/417). In the univariable analysis, older age, hypertension, and low preoperative eGFR were significantly associated with postoperative delirium in the old age group. However, there was no significant association between intraoperative reduction in rSO_2_ and postoperative delirium for all cut-offs in either the univariable or the multivariable logistic regression analysis.

## Discussion

The results of this study suggest that decreases in intraoperative rSO_2_ below 50% are associated with postoperative delirium after OPCAB. This was also associated with postoperative acute kidney injury and longer ICU and hospital stays. Among patients less than 68 years of age, rSO_2_ lower than 55% was associated with postoperative delirium. However, in patients more than 68 years old, intraoperative rSO_2_ was not associated with postoperative delirium.

The incidence of delirium in this study was 12.9%, slightly lower than reported by previous studies using similar diagnostic methods (23 to 52%) [9]. One of the reasons for this difference may be the age of the included patients, half of whom were under 68 years of age. Conversely, previous studies have included mostly patients over 60 years of age [9]. Age is one of the most powerful risk factors for delirium after cardiac surgery [29]. Furthermore, we selected only patients who had underwent OPCAB, while in previous studies both on-pump and off-pump cardiac surgery were included, with on-pump surgery being more common [9, 18, 19, 29]. Although the topic remains controversial, some studies have suggested that beating heart surgery can lower the risk of delirium caused by solid microemboli or the alteration of cerebral autoregulation during the cardiopulmonary bypass (CPB) period [18, 23, 30].

Considering the cut-off values for intraoperative rSO_2_ during cardiac surgery, Yao and colleagues [17] set multiple thresholds indicating different degrees of hypoxic brain injury. They used 50, 45, 40, 35, and 30% as absolute values, corresponding to the baseline value minus 1, 1.5, 2, 2.5, and 3 SDs. A rSO_2_ reduction below 40% was significantly associated with postoperative neurologic dysfunction after cardiac surgery with CPB based on a multivariable analysis. In several studies, including randomized controlled trials, prolonged cerebral desaturation below 50% as an absolute value or more than 20% of baseline was associated with postoperative cognitive decline [31–34]. However, these studies were mostly conducted on cardiac surgery with CPB and evaluated only one or two thresholds rather than various cut-off ranges.

We aimed to determine whether there is a certain cut-off value for intraoperative rSO_2_ during OPCAB associated with increased postoperative delirium. Previously, it has been shown that rSO_2_ values measured by cerebral oximetry reflect a balance between oxygen consumption and supply in the frontal lobe, especially in the “water-shed” area in the junction between the anterior and middle cerebral arteries [3, 16]. Intraoperative cerebral hypoperfusion is also known to be related to postoperative neurological dysfunction after cardiac surgery [17, 30–32]. However, several randomized controlled trials showed inconsistent results regarding the relationship between intraoperative rSO_2_ reductions during cardiac surgery and postoperative neurologic outcomes. Two meta-analyses focusing on the use of cerebral oximetry and postoperative outcomes after cardiac surgery concluded that there was a low level of evidence linking intraoperative reductions in rSO_2_ with postoperative neurologic outcomes [13, 35].

There may be several reasons for the inconsistent results regarding the usefulness of cerebral oximetry during cardiac surgery. First, heterogeneous patients were enrolled in previous studies. These studies involved various types of cardiovascular surgeries, including valvar surgery, coronary artery bypass surgery, cardiac tumor surgery, and aortic surgery, which involve different applications of intraoperative CPB and hypothermia. Transient but significant dysfunction in cerebral autoregulation and cerebral desaturation due to hemodilution or microemboli may occur with CPB. Cerebral oxygen consumption is also altered during CPB and hypothermia [14, 15, 17, 18, 36, 37]. Thus, with or without CPB, these heterogeneous populations may have led to inconsistent results. In the current study, to increase the homogeneity of patients, we included only patients who had undergone OPCAB without CPB.

In addition, previous studies including several randomized controlled trials, used various protocols and rSO_2_ cut-off values to trigger intervention to restore rSO_2_. This may also have contributed to the inconsistent results. Conversely, we evaluated the relationship between rSO_2_ reductions and postoperative delirium at various cut-off values. By analyzing not only the occurrence but also the total duration of rSO_2_ reduction, we aimed to identify the threshold of hypoxia exceeding the compensating capacity of the brain relating to the duration of cerebral desaturation.

We also included intraoperative MAP, C.I., and SvO_2_ as risk factors for postoperative delirium occurrence. Although these hemodynamic variables can affect intraoperative cerebral perfusion and consequently postoperative delirium, they have not been included in many previous studies. In our study, by conducting a regression analysis, we attempted to rule out the possibility of multicollinearity between these hemodynamic variables and rSO_2_.

In the subgroup analysis of patients under age 68, only preoperative EF, level of albumin, and C-reactive protein were associated with postoperative delirium by univariable analysis. The cut-off value of rSO_2_ associated with postoperative delirium was 55%, which was slightly higher than the 50% cut-off for the entire study group. Moreover, in patients over age 68, rSO_2_ was not associated with postoperative delirium. The pathophysiology of postoperative delirium is complex, and age is one of the most powerful risk factors, along with history of hypertension [6, 9, 19, 21]. Thus, in old patients, other factors associated with old age may more strongly influence the occurrence of postoperative delirium than intraoperative brain oxygenation.

This study has several limitations. First, because this study was retrospective in nature, risk factors that could affect postoperative delirium could not be perfectly controlled. Similarly, the anesthetic management to maintain or restore rSO_2_ was not controlled. Second, this study involved cardiac surgery cases from 2004 to 2016, and surgical and anesthetic methods and techniques evolved over this period. These changes may have influenced the occurrence of postoperative delirium. Third, preoperative neurologic function was not assessed, and postoperative delirium was estimated using medical records and prescription history. The incidence of postoperative delirium may therefore have been underestimated. Finally, we could not assess the baseline rSO_2_ values. Previous studies consistently found that preoperative baseline rSO_2_ was associated with postoperative delirium in cardiac surgery [13, 15, 35]. However, since this was a retrospective study we could not access or identify the baseline rSO_2_ before anesthesia induction or at the beginning of the surgery, and also the impact of baseline rSO_2_ on postoperative delirium could not be evaluated. Consequently, the decrease in rSO_2_ relative to the baseline was not estimated. The differences between the patients who were with low baseline and lesser reduction of rSO_2_ and with high baseline and more reduction of rSO_2_, could not be evaluated. We suggested that the baseline value itself may not be in normal physiologic values for cardiac surgery patients and the reserves from the baseline values may be different by the individuals. We based our hypothesis on our clinical experience, and thus we considered absolute cut-offs to be of more clinical significance. Considering the limitations of this study, prospective, randomized controlled studies may be needed to evaluate the effect of intervention to maintain rSO_2_ over 50% (or 55% for patients under 68 years of age) during OPCAB.

## Conclusions

In patients undergoing OPCAB, intraoperative rSO_2_ below 50% was associated with postoperative delirium. Among patients younger than 68 years old, rSO_2_ below 55% was associated with postoperative delirium. Therefore, rSO_2_ should be maintained at over 50%, or over 55% among patients less than 68 years old, during OPCAB.

## Supplementary information

**Additional file 1 **revised.docx **Supplementary tables.**

**Additional file 2 **JPG **Supplementary Fig. 1** The ROC curves of multivariable prediction model for patients under age 68

## Data Availability

The datasets used and/or analyzed during the current study are available from the corresponding author on reasonable request.

## Abbreviations

NIRS: Near-infrared spectroscopy
rSO_2_: Regional cerebral oxygen saturation
OPCAB: Off pump coronary artery bypass graft surgery
CABG: Coronary artery bypass graft surgery
SD: Standard deviation
SvO_2_: Mixed venous oxygen saturation
C.I.: Cardiac index
ICU: Intensive care unit
EF: Ejection fraction
eGFR: Estimated glomerular filtration rate
MAP: Mean arterial pressure
ROC: Receiver operating characteristic
CAM: Confusion assessment method
DSM-5: Diagnostic and statistical manual of mental disorders-5
ASA: American society of anesthesiologists
OR: Odds ratio
CI: Confidence interval
AUROC: Area under receiver operating characteristic
CPB: Cardiopulmonary bypass
