# Supplementary material for: The relationship between intraoperative cerebral oximetry and postoperative delirium in patients undergoing off-pump coronary artery bypass graft surgery: a retrospective study
Source: BMC Anesthesiol. 2020 Nov 14;20:285. doi: 10.1186/s12871-020-01180-x (PMC7666484; doi:10.1186/s12871-020-01180-x)
Supplement: Supplementary file 1 — Additional file 1 revised.docx Supplementary tables. [file 12871_2020_1180_MOESM1_ESM.docx]

| **Supplementary Table 1** Comparison of intraoperative hemodynamic variables between delirium and no delirium group | | | |
| --- | --- | --- | --- |
| Hemodynamic variables | No delirium (n=710) | Delirium (n=105) | *P*-values |
| MAP |  |  |  |
| Mean duratin of reduction < 68mmHg (min) | 128.5±112.8 | 169.5±134.8 | 0.001 |
| Minimum (mmHg) | 54.2±7.6 | 52.5±6.5 | 0.03 |
| C.I. |  |  |  |
| Mean duration of reduction < 2.2L/min/m^2^ (min) | 181.9±158.9 | 240.2±180.4 | 0.001 |
| Minimum (L/min/m^2^) | 1.7±0.3 | 1.6±0.3 | 0.05 |
| SvO_2_ |  |  |  |
| Mean duration of reduction < 64% (min) | 50.7±91.1 | 90.6±146.4 | 0.001 |
| Minimum (%) | 62.3±7.4 | 60.5±8.9 | 0.03 |
| The values are expressed as mean (standard deviation)  *MAP* mean arterial pressure, *C.I.* cardiac index, *SvO_2_* mixed venous oxygen saturation | | | |

| **Supplementary Table 2** The relationship between intraoperative rSO_2_ and postoperative AKI, ICU and hospital length of stay. | | | | | | |
| --- | --- | --- | --- | --- | --- | --- |
|  | No AKI (n=648) | AKI (n=167) | *P-*values | Pearson correlation | ICU_LOS 2.8±3.24 (d) | Hospital_LOS 11.5±11.96 (d) |
| rSO_2_ (%) |  |  |  | rSO_2_ |  |  |
| Mean | 56.2(6.3) | 52.3(8.2) | <0.001 | Mean | -0.07* | -0.08* |
| Minimum | 48.1(7.8) | 45.0(8.6) | <0.001 | Minimum | -0.09* | -0.048 |
|  |  |  |  |  |  |  |
| Mean duration of rSO2 reduction (min) | | | | Mean duration of rSO2 reduction | | |
| <50% | 88.3±152.4 | 173.5±197.5 | <0.001 | <50% | 0.15** | 0.12** |
| <45% | 30.6±85.9 | 88.9±156.9 | <0.001 | <45% | 0.20** | 0.14** |
| For AKI, the values are expressed as mean (standard deviation) and for LOS, Pearson correlations between two values  *rSO_2_* regional cerebral oxygen saturation, *AKI* acute kidney injury, *ICU* intensive care unit, *LOS* length of stay  * *P*-value<0.05, ** *P*-value <0.01 | | | | | | |

| **Supplementary Table 3** Baseline and perioperative characteristics of patients age under 68 | | | |  |
| --- | --- | --- | --- | --- |
| Characteristics | No delirium (n=379) | Delirium (n=19) | *P*-values | |
| Patients characteristics |  |  |  | |
| Age (year) | 58.3±7.3 | 58.4±6.6 | 0.96 | |
| Male sex | 312(82.3%) | 16(84.2%) | 0.83 | |
| BMI (kg.m^-2^) | 25.0±3.4 | 25.3±3.3 | 0.73 | |
| Preoperative medical status |  |  |  | |
| ASA physical status |  |  | 0.69 | |
| 1 | 11(2.9%) | 0 |  | |
| 2 | 118(31.1%) | 7(36.8%) |  | |
| 3 | 244(64.4%) | 11(57.9%) |  | |
| 4 | 6(1.6%) | 1((5.3%) |  | |
| Hypertension | 228(60.2%) | 14(73.7%) | 0.25 | |
| Diabetes mellitus | 198(52.2%) | 12(63.2%) | 0.36 | |
| Dyslipidaemia | 143(37.7%) | 9(47.4%) | 0.40 | |
| Myocardial infarction | 45(11.9%) | 3(15.8%) | 0.61 | |
| Atrial fibrillation | 23(6.1%) | 1(5.3%) | 0.89 | |
| Chronic kidney disease | 142(37.5%) | 10(52.6%) | 0.19 | |
| History of stroke | 238(62.8%) | 9(47.4%) | 0.18 | |
| Left ventricle ejection fraction (%) | 56.6±11.5 | 49.7±13.9 | 0.08 | |
| Haematocrit (%) | 35.1±4.0 | 33.8±3.7 | 0.17 | |
| Creatinine (mg/dL) | 1.5±2.0 | 2.0±2.6 | 0.26 | |
| estimated GFR (ml/min/1.73/m^2^) | 75.2±28.6 | 69.7±32.7 | 0.33 | |
| Albumin (g/dL) | 4.1±0.4 | 3.9±0.4 | 0.04 | |
| C-reactive protein (mg/dL) | 0.6±1.4 | 1.8±4.0 | 0.02 | |
| **Intraoperative variables** |  |  |  | |
| Operation duration (min) | 365.0±53.8 | 371.0±58.6 | 0.64 | |
| Re-do operation | 1(0.3%) | 0 | 1 | |
| Emergency | 38(10.0%) | 2(10.5%) | 0.94 | |
| **MAP**  Mean (mmHg) | 74.7±5.5 | 73.8±5.8 | 0.50 | |
| Minimum (mmHg) | 55.4±7.4 | 56.0±9.4 | 0.74 | |
| **C.I.** |  |  |  | |
| Mean (L/min/m^2^) | 2.4±0.4 | 2.4±0.4 | 0.94 | |
| Minimum (L/min/m^2^) | 1.7±0.3 | 1.8±0.4 | 0.88 | |
| **SvO2** |  |  |  | |
| Mean (%) | 71.9±5.6 | 71.0±6.5 | 0.50 | |
| Minimum (%) | 63.0±7.15 | 61.8±8.06 | 0.49 | |

The values are expressed as mean (standard deviation) and number (%) for categorized variables

| *BMI* body mass index, *GFR* glomerular filtration rate, *MAP* mean arterial pressure, *C.I.* cardiac index, *SvO_2_* mixed venous oxygen saturation  **Supplementary Table 4** Comparison of intraoperative rSO_2_ between delirium and no delirium group in age under 68 | | | |
| --- | --- | --- | --- |
| rSO_2_ | No delirium (n=379) | Delirium (n=19) | *P*-values |
| Mean (%) | 55.60±7.3 | 50.6±7.7 | 0.004 |
| Minimum (%) | 47.7±8.2 | 42.7±7.4 | 0.01 |
|  |  |  |  |
| Mean duration of rSO_2_ reduction (min) | |  |  |
| <75% | 450.5±141.7 | 445.0±122.9 | 0.87 |
| <70% | 438.9±151.1 | 441.3±126.2 | 0.95 |
| <65% | 394.6±172.0 | 422.9±136.2 | 0.48 |
| <60% | 316.4±198.7 | 381.6±169.3 | 0.16 |
| <55% | 207.1±196.5 | 311.8±196.6 | 0.02 |
| <50% | 102.5±159.7 | 219.7±200.5 | 0.002 |
| <45% | 43.5±110.3 | 114.0±162.4 | 0.01 |
| <40% | 12.4±54.7 | 26.8±94.6 | 0.29 |
| <35% | 5.3±37.7 | 14.0±60.8 | 0.35 |
|  |  |  |  |
| Number of patients with rSO_2_ reduction | |  |  |
| <70% | 378(99.7%) | 19(100%) | 1 |
| <65% | 375(98.9%) | 19(100%) | 1 |
| <60% | 355(93.7%) | 19(100%) | 0.26 |
| <55% | 297(78.4%) | 18(94.7%) | 0.09 |
| <50% | 213(56.2%) | 16(84.2%) | 0.02 |
| <45% | 130(34.3%) | 11(57.9%) | 0.04 |
| <40% | 56(14.8%) | 6(31.6%) | 0.049 |
| <35% | 18(4.7%) | 1(5.3%) | 0.92 |
| The values are expressed as mean (standard deviation) for baseline, mean, minimum rSO_2_ and mean duration of rSO_2_ reduction, number (%) for the incidence of rSO_2_ reduction  *rSO_2_* regional cerebral oxygen saturation | | | |
