# Supplementary figures and images for: The relationship between intraoperative cerebral oximetry and postoperative delirium in patients undergoing off-pump coronary artery bypass graft surgery: a retrospective study
Source: BMC Anesthesiol. 2020 Nov 14;20:285. doi: 10.1186/s12871-020-01180-x (PMC7666484; doi:10.1186/s12871-020-01180-x)

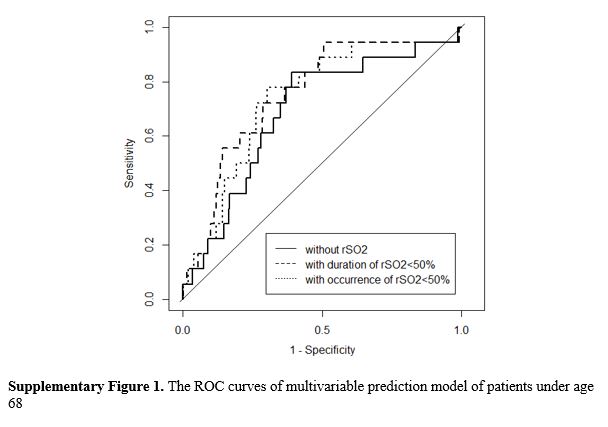

Supplement: Supplementary file 2 — Additional file 2 JPG Supplementary Fig. 1 The ROC curves of multivariable prediction model for patients under age 68 [file 12871_2020_1180_MOESM2_ESM.jpg]
